# DSTYK Promotes Metastasis and Chemoresistance *via* EMT in Colorectal Cancer

**DOI:** 10.3389/fphar.2020.01250

**Published:** 2020-09-02

**Authors:** Jinyu Zhang, Zachary Miller, Phillip R. Musich, Ashlin E. Thomas, Zhi Q. Yao, Qian Xie, Philip H. Howe, Yong Jiang

**Affiliations:** ^1^Department of Biomedical Sciences, J. H. Quillen College of Medicine, East Tennessee State University, Johnson City, TN, United States; ^2^Division of Infectious, Inflammatory and Immunologic Diseases, Department of Internal Medicine, Quillen College of Medicine, ETSU, Johnson City, TN, United States; ^3^Department of Biochemistry and Molecular Biology, Medical University of South Carolina, Charleston, SC, United States

**Keywords:** dual serine/threonine and tyrosine protein kinase, colorectal cancer, epithelial-mesenchymal transition, transforming growth factor-β, chemoresistance, metastasis

## Abstract

**Objective:**

Tumor metastasis and resistance to chemotherapy are two critical factors that contribute to the high death rate of colorectal cancer (CRC) patients. Metastasis is facilitated by the epithelial-mesenchymal transition (EMT) of tumor cells, which has emerged not only as a fundamental process during metastasis, but is also a key process leading to chemoresistance of cancer cells. However, the underlying mechanisms of EMT in CRC cell remain unknown. Here, we aim to assess the role of dual serine/threonine and tyrosine protein kinase (DSTYK) in CRC metastasis and chemoresistance.

**Methods:**

To study the role of DSTYK in TGF-β-induced EMT, we employed techniques including Crispr/Cas9 knockout (KO) to generate DSTYK KO cell lines, RT-PCR to detect the mRNA expression, immunofluorescence analyses, and western blots to detect protein levels of DSTYK in the following 4 cell lines: control LS411N-TβRII and LS411N-TβRII/DSTYK KO, control LS513 and LS513/DSTYK KO cells, treated with/without TGF-β. The effects of DSTYK on apoptosis were investigated by MTT assays, flow cytometry assays, and TUNEL assays. The expression of DSTYK in CRC patients and its correlation with EMT markers were determined by bioinformatics analysis. For *in vivo* analysis, both xenograft and orthotopic tumor mouse models were employed to investigate the function of DSTYK in chemoresistance and metastasis of tumors.

**Results:**

In this study, we demonstrate that the novel kinase DSTYK promotes both TGF-β-induced EMT and the subsequent chemoresistance in CRC cells. DSTYK KO significantly attenuates TGF-β–induced EMT and chemoresistance in CRC cells. According to the Gene Expression Omnibus (GEO) database, the expression of DSTYK is not only positively correlated to the expression of TGF-β, but proportional to the death rate of CRC patients as well. Evidently, the expression of DSTYK in the metastatic colorectal cancer samples from patients was significantly higher than that of primary colorectal cancer samples. Further, we demonstrate in mouse models that chemotherapeutic drug treatment suppresses the growth of DSTYK KO tumors more effectively than control tumors.

**Conclusion:**

Our findings identify DSTYK as a novel protein kinase in regulating TGF-β–mediated EMT and chemoresistance in CRC cells, which defines DSTYK as a potential therapeutic target for CRC therapy.

## Introduction

Colorectal cancer (CRC) is the third leading cause of cancer death worldwide; the 5-year relative survival rate is only 53% to 65% in spite of many efforts to improve diagnosis and chemotherapy (Torre et al., 2015). Currently, tumor metastasis and resistance to chemotherapeutic treatment are considered as two critical factors that contribute to the death of CRC patients (Tsoumas et al., 2018). Approximately 25% of newly diagnosed CRC patients develop metastases and 50% of all CRC patients die from metastatic disease (Obrand and Gordon, 1997). The process of epithelial–mesenchymal transition (EMT) has been shown to play a critical role in promoting metastasis in epithelium-derived carcinoma (Thomson et al., 2005). Metastasis is initiated through the EMT of tumor cells, in which cells undergo a switch from a polarized, epithelial phenotype to a highly motile and invasive fibroblastic or mesenchymal phenotype (Singh and Settleman, 2010). EMT has emerged not only as a fundamental process during normal embryonic development (Lim and Thiery, 2012) and in adult tissue homeostasis (Antonello et al., 2015), but it also is aberrantly activated during the metastatic progression in cancer (Tsai and Yang, 2013; Aiello et al., 2017; Lu and Kang, 2019). In addition to metastasis, EMT is strongly associated with chemoresistance upon chemotherapeutic treatment. Tumor cells with the capacity of chemoresistance usually show a strong mesenchymal phenotype (Shah et al., 2007) and *vice versa*, tumor cells undergoing EMT also acquire the capability of chemoresistance (Yin et al., 2007). A number of cytokines and their signaling pathways have been shown to regulate EMT, such as transforming growth factor-β (TGF-β), Wnts, Notch, fibroblast growth factors (FGFs) and bone morphogenetic protein (BMP), etc. Preventing EMT will increase significantly the drug sensitivity of tumor cells and the survival rate of human CRC patients (Ren et al., 2013; Fischer et al., 2015; Zheng et al., 2015). TGF-β is an established inducer of EMT; however, it remains unclear how this signaling pathway induces EMT and whether there are other novel signaling pathways or elements involved in EMT (Xu et al., 2009). Therefore, the potential for improving chemotherapeutic efficacy in CRC patients critically depends on improving our understanding of the mechanism by which TGF-β induces EMT in CRC cells leading to chemoresistance and metastasis (Goldberg et al., 2004; Goldberg et al., 2006).

DSTYK is a dual serine/threonine and tyrosine protein kinase expressed in multiple human tissues including brain, heart, kidney, lung, colon, and muscle (Peng et al., 2006). The functions of DSTYK are barely known, although it has been reported that mutations of DSTYK are associated with hereditary spastic paraplegia type 23 (Lee et al., 2017). Also, DSTYK has been shown to play a predominant role in suppressing caspase-dependent apoptosis caused by ultraviolet (UV) light exposure in skin cells (Lee et al., 2017). However, the role of DSTYK in CRC metastasis and chemoresistance remains unknown. To address this question, we characterized the function of DSTYK not only in a cellular model, but also in a xenograft mouse model. We further evaluated the role of DSTYK in CRC chemoresistance and metastasis with an orthotopic mouse model.

In this study, we sought to investigate the biological function of DSTYK in CRC. We found that DSTYK facilitates TGF-β-induced EMT and promotes chemoresistance in two CRC cell lines, LS411N-TβRII and LS513, by inhibiting apoptosis. Additionally, we evaluated DSTYK as a novel therapeutic target for CRC therapy in mouse models. The reported findings will increase our understanding of the function of DSTYK in CRC and will enhance our ability in developing treatments to prevent both EMT and chemoresistance. Thus, our findings present DSTYK as a highly promising therapeutic target to significantly improve CRC treatment.

## Materials and Methods

### Cell Culture and Reagents

Human colorectal cancer cell lines LS411N and LS513 were purchased from the American Type Culture Collection (ATCC, Manassas, VA, United States), and the LS411N-TβRII cell line was generated in our lab by overexpressing TGF-β receptor II (TβRII) in LS411N cells. All cells were cultured in RPMI-1640 medium containing 10% FBS and 1% antibiotic and antimycotic cocktail (Millipore). The cells were maintained at 37°C with 5% CO_2_. The culture medium was refreshed two or three times a week. TGF-β signaling pathway inhibitor LY2109761 was purchased from MedChemExpress LLC.

### Annexin V-FITC/PI Double-Staining Assay

Cells were seeded in 12-well plates and incubated overnight for attachment, then the media was replaced with new media for either TGF-β or oxaliplatin (OXA) treatment. After treatment, all floating and adherent cells were collected by trypsinization. All cells were collected by centrifugation and washed three times in phosphate-buffered saline (PBS) before staining with Annexin V-FITC and PI according to the specified protocol (Cell Signaling Technology, cat# 6592). Briefly, cells were resuspended in 500 µl 1× binding buffer containing 5 µl Annexin V-fluorescein isothiocyanate (FITC) and 10 µl propidium iodide (PI) in the dark for 5 min. The stained cells were analyzed with a flow cytometer and the data were analyzed using FlowJo cell software (FlowJo, LLC, Ashland, Ore) to measure apoptotic cells, live cells, and necrotic cells. Early apoptosis was designated as annexin V positive/PI negative and late apoptosis was designated as annexin V positive/PI positive; necrosis was defined as annexin V negative/PI positive.

### MTT Assay and Clonogenic Assay

The MTT assay was performed as previously described (Jiang et al., 2016). Briefly, 5,000 cells per well of LS411N-TβRII or LS513 cells were seeded in a 96-well dish with 200 μl RPMI containing 10% FBS and incubated in a 37 °C and 5% CO2 incubator. Post-plating (24 h), various concentrations of OXA or vehicle were added. Following a 48 h treatment, cell viability was assessed by the MTT (3-[4, 5-dimethylthiazol-2-yl]-2, 5-diphenyl tetrazolium bromide; Sigma) assay according to the manufacturer’s protocol. The viable fraction is expressed as the percentage of vehicle-treated control cells. For the clonogenic assay, the same number of cells were seeded into each well in 6-well plates. After drug treatment for 2 days, the media were changed and the cells were continuously cultured for additional 7 to 10 days in a drug-free medium and followed by fixation and staining with crystal violet and photography.

### Western Blotting

Western blot (WB) analyses were performed as previously described (Jiang et al., 2016). Total proteins were separated by loading a specified amount of whole cell lysate on a denaturing 8% to 15% SDS-polyacrylamide gel followed by transferring to polyvinylidene difluoride (PVDF) membranes. Membranes were blocked with a 5% non-fat milk solution and incubated with primary antibodies for vimentin, Bax, Bcl-2, Bim (Cell Signaling Biotechnology), E-cadherin (BD Biosciences), N-cadherin (BD Biosciences), DSTYK (Thermo Fisher Scientific) or Hsp90 (Santa Cruz Biotechnology). Secondary antibodies (Thermo Fisher Scientific) conjugated to horseradish peroxidase and ECL (Bio-rad) were used to detect signals that were visualized by chemiluminescence. At least three independent experiments were performed for each western blotting assay.

### Real Time-Quantitative PCR (RT-qPCR)

Total mRNA was extracted by using the Oligotex Direct mRNA Mini Kit (Qiagen) and cDNA was synthesized from 1 μg of mRNA by using High-Capacity cDNA Reverse Transcription Kits (Bio-Rad) plus SYB^®^ Gene Expression Assay Mix, sterile water, and Fast Universal PCR Master Mix (Applied Biosystems) to measure specific gene expression. Real-time PCR was carried out using the CFX Real-Time PCR Detection system (Bio-Rad). The expression of a target gene was calculated relative to the expression of β-actin.

### Terminal Deoxynucleotidyl Transferase-Mediated dUTP Nick End-Labeling (TUNEL) Assay

The TUNEL assay was performed as described in the supplier’s protocol from ABP Biosciences (catalog number: A050). Briefly, cells were cultured on coverslips in 24-well plates and fixed in 4% paraformaldehyde in PBS. The TdT reaction was performed for 1 h at 37 °C in a humidified chamber. Then, the cells were stained with 4, 6-diamidino-2-phenylindole (DAPI) for 5 min. Fluorescence signal was examined, and photos were taken with a Leica fluorescence microscopy.

### Immunofluorescence Staining

For immunofluorescence, cells cultured on coverslips were fixed in 4% paraformaldehyde for 10 min, permeabilized with 0.1% Triton X-100 for 15 min and blocked with 3% BSA (all in PBS) for 30 min. Cells then were incubated sequentially at 4°C overnight with primary antibodies and then with Alexa Fluor 488-labeled secondary antibodies (Abcam). Nuclei were counterstained with DAPI (Vector Laboratories, 1:500). Confocal fluorescence images were taken with a Leica TCS SP8 confocal microscope.

### DSTYK Knockout (KO) *via* Crispr/Cas9

Cells were transfected with DSTYK Crispr/Cas9 KO Plasmid (Santa Cruz, sc-404521) and HDR plasmids (Santa Cruz, sc-404521-HDR) using lipofectamine 3000 (ThermoFisher Scientific) according to the manufacturer’s instructions. After transfection for 48 h, the cells were cultured in puromycin-containing media for selection and cloning of DSTYK knockout (KO) cells. DSTYK KO was confirmed by WB analysis.

### Xenograft and Metastasis Experiments in Mice

All animal experimental procedures are consistent with our mice protocols that were approved by the Institutional Animal Care and Use Committee at East Tennessee State University (ETSU). NOD.CB17-Prkdcscid/J mice (SCID), 4–6 weeks old, were purchased from the Jackson laboratory and were maintained at our animal facility under the specified pathogen-free condition. In all experiments, male mice were randomly selected. In the xenograft mouse model, 2 × 10^5^ LS411N-TβRII cells or 2 × 10^5^ LS513 cells were injected into the flanks of 7- to 8-week-old SCID mice. When the tumor volume reached about 50 mm^3^, mice received therapy by intraperitoneal (i.p.) injection of the chemotherapeutic agent oxaliplatin (OXA). OXA (10–20 mg/kg) was administered to mice 2 times/week intraperitoneally for 3–4 weeks. Finally, mice were euthanized by carbon dioxide, subjected to necropsy, and tissues collection. Tumors were measured every 2 to 3 days using calipers and calculated using a standard formula: width^2^ × length × 0.52. Body weights were measured weekly.

In the orthotopic metastatic models, 5 × 10^4^ cells (LS411N-TβRII or LS513) were injected into the cecum wall of mice at the age of 8 weeks. When signs of disease were visible, mice were euthanized by carbon dioxide, subjected to necropsy, and inspected for tumors in cecum, liver or lung. Primary carcinomas in the cecum and metastases in the livers were collected for histological analysis. Each study was repeated three times unless otherwise specified.

### Tumor Tissues From Patients

Specimens [patients (n=13), normal, pre-tumor, tumor, metastasis] as shown in **Supplemental Table S1** were obtained from patients who had undergone CRC resection and with tumor grade categorized as well differentiated, moderately differentiated, poorly differentiated, or undifferentiated by three pathologists. Informed consent was obtained from all patients before surgery, and the use of histological sections was approved by our ethics review committee.

### Immunohistochemistry

Paraffin-embedded tissue sections (5 µm thick) were stained with anti-DSTYK antibody, anti-cleaved caspase 3 antibody (1:100, Cell Signaling Biotechnology) or anti–Ki-67 antibody (1:100, Cell Signaling Biotechnology) overnight at 4°C. The signals were detected using the Vectastain Elite ABC kit (Vector Laboratories, Burlingame, CA) following the manufacturer’s protocol. Hematoxylin was used for counterstaining. The signal intensity was scored using the following scale: 0 (negative), 1 (very weak), 2 (weak), 3 (moderate), 4 (middle strong), 5 (strong), and 6 (very strong). The staining was considered to be positive if the sum of distribution and intensity scores was greater than 2. Tissue microarray (TMA) blocks were prepared for pre-metastatic and metastatic colorectal cancer samples, primary colorectal cancer samples, and paired normal colon counterparts, following our above immunohistochemistry protocol.

### Statistical Analysis

All results are expressed as the mean ± SD or SEM. Mice and sample groups were indicated. Data were analyzed using the unpaired student t-test, and treatment differences were considered significant at P value < 0.05. Kaplan-Meier survival curves were generated using GraphPad Prism software.

## Results

### DSTYK Is Upregulated in Human CRC Metastatic Progression

**Figure 1:**
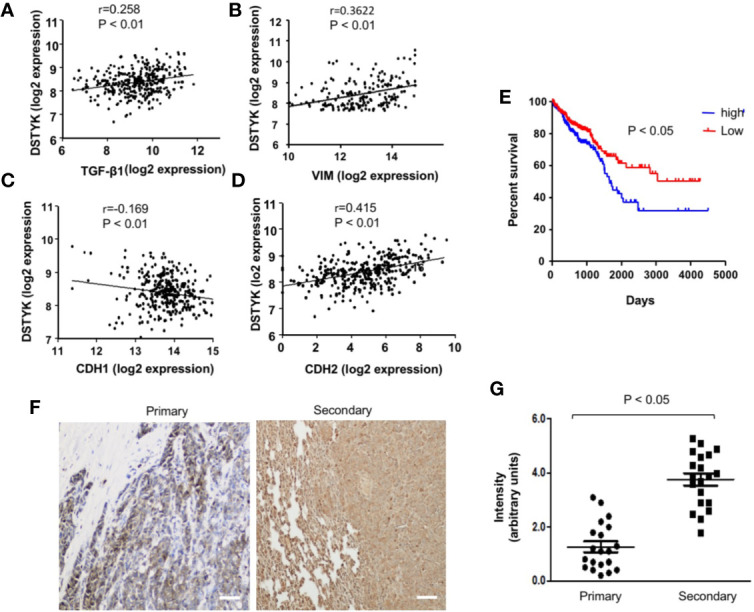
DSTYK is associated with the metastasis of human colorectal cancer. **(A)** Scatter plot showing the correlation between the mRNA levels of DSTYK and TGF-β, **(B)** DSTYK and vimentin (VIM), **(C)** DSTYK and E-cadherin (CDH1), and **(D)** DSTYK and N-cadherin (CDH2) in the human TCGA colon cancer database. **(E)** Kaplan-Meier curve assessing the correlation between DSTYK expression and patients’ survival rate obtained from 652 patients in The Gene Expression Omnibus (GEO) database. Blue line represents metastatic colorectal cancer patients with high DSTYK expression (n=154) and the red line represents patients with low DSTYK expression (n=498) in the GSE 17538 dataset. SPSS repeated measures, general linear model. **(F)** Representative images of human colorectal primary tumors and secondary tumors subjected to immunohistochemical (IHC) analysis with DSTYK antibody. Images were taken after IHC staining. Scale bar, 50 µm. **(G)** Quantification of the staining signals from IHC analyses of DSTYK in both primary and secondary tumor tissues. Data are presented as means ± SEM. for n= 20.

From our previous gene microarray analysis, we found that the expression of DSTYK is significantly higher in TGF-β-induced EMT in CRC cells. To further investigate the role of DSTYK in the progression of human CRC, we analyzed the data of DSTYK mRNA expression in The Gene Expression Omnibus (GEO) database of the human CRC patients. DSTYK expression is positively proportional to the expression of TGF-β, mesenchymal markers vimentin (VIM) and N-cadherin (CDH2) (**Figures 1A, B, D**) in tumor tissues from human CRC patients, whereas it is inversely correlated to epithelial marker E-cadherin (CDH1) expression (**Figure 1C**). These findings imply that DSTYK may be involved in regulating EMT. We also found that higher levels of DSTYK are correlated with poor prognosis of colorectal cancer patients as indicated by the inversely proportional correlation between the level of DSTYK and the survival rate of CRC patients (**Figure 1E**). In addition, we performed immunohistochemical (IHC) staining of 20 primary human colorectal tumors and 20 secondary tumors (metastases from primary colorectal tumors) including those in lungs and livers (normal, pre-tumor, tumor, metastasis as shown are from patients listed in **Supplemental Table 1** (**Figure 1F**). The quantified results showed that the mean signal of DSTYK in secondary tumors is significantly higher than that in primary tumors (**Figure 1G**), which implies that DSTYK plays an important role in CRC metastasis. All these data suggest that DSTYK may contribute to human CRC metastasis.

### TGF-β Induces DSTYK Expression and EMT in Colorectal Cancer Cells

**Figure 2:**
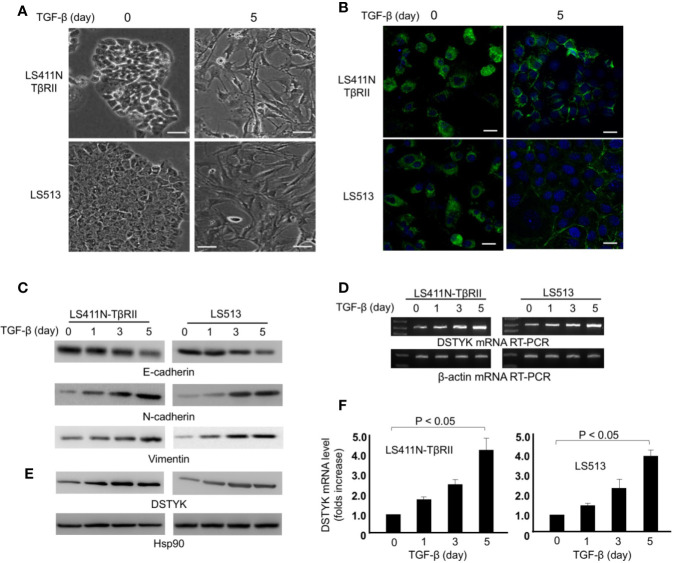
TGF-β induces EMT in LS411N-TβRII cells and LS513 cells. **(A)** Morphological change of cells before and after 5 days of TGF-β (1 ng/ml) treatment. Scale bar: 100 µm. **(B)** Representative immunostaining of E-cadherin in LS411N-TβRII cells and LS513 cells before and after 5 days TGF-β (1 ng/ml) treatment. Scale bar, 50 μm. **(C)** WB analyses to detect the protein levels of epithelial marker E-cadherin and mesenchymal markers, N-cadherin and vimentin. Hsp90 was used as loading control. All experiments were repeated at least three times and similar results were observed. **(D)** RT-PCR analyses of DSTYK mRNA levels during TGF-β-induced EMT. β-actin was used as control. **(E)** Immunoblotting analysis to detect the protein levels of DSTYK during TGF-β-mediated EMT. Hsp90 was used as loading control. All experiments were repeated at least three times and similar results were observed. **(F)** Real-time PCR analyses to detect the upregulation of DSTYK expression during TGF-β-mediated EMT. Data are shown as mean ± SD, n=3 independent experiments.

To validate that DSTYK is involved in TGF-β-induced EMT in CRC, we used two cell lines, LS411N-TβRII and LS513. Because LS411N cells have a defective TGF-β receptor II (TβRII) and are not responsive to TGF-β treatment (de Miranda et al., 2015), we ectopically expressed wild-type (WT) TβRII in LS411N cells. After WT TβRII was introduced, LS411N cells became TGF-β competent and were defined as LS411N-TβRII cells. The specific effects of the TGF-β signaling pathway can be warranted in experiments performed with this cell line. LS513 is another TGF-β competent cells line being used as a parallel control. LS411N-TβRII cells and LS513 cells were treated with TGF-β1 (1 ng/ml) for 5 days to induce EMT. During this 5-day treatment, both cells showed a morphology change from the cobblestone-like epithelial cells to the fibroblast-like cells (**Figure 2A**). Whole cell lysates from TGF-β-treated cells were subjected to immunoblotting analysis to detect the expression of epithelial cell marker E-cadherin, and mesenchymal markers, N-cadherin, and vimentin. The amount of E-cadherin was significantly decreased in cells treated with TGF-β, which is indicated by both immunofluorescence and immunoblotting assays (**Figures 2B, C**), whereas the amounts of N-cadherin and vimentin were significantly increased in those cells with TGF-β treatment when compared to control (**Figure 2C**). These findings support the conclusion that EMT is induced by TGF-β. Further, we determined whether the level of DSTYK mRNA is modified upon TGF-β treatment. We performed both reverse transcription PCR (**Figure 2D**) and real-time PCR with mRNA extracts from the indicated time course with TGF-β treatment (**Figure 2F**). The results from both PCR analyses indicate that DSTYK message is upregulated during this EMT induction. Furthermore, immunoblotting analyses showed that both LS411N-TβRII cells and LS513 cells carry a low level of DSTYK proteins and DSTYK protein levels were significantly increased during TGF-β-mediated EMT in both cell lines (**Figure 2E**). To confirm that an intact TGF-β signaling pathway is required for DSTYK upregulation, we employed a commercially available TGF-β signaling inhibitor, LY2109761, in both LS411N-TβRII cells and LS513 cells. The results show that TGF-β has no effect on DSTYK levels in the presence of the TGF-β signaling inhibitor (**Supplemental Figures 1A, B**). Also, we did not find any TGF-β treatment-induced upregulation of DSTYK in LS411N cells in which the endogenous mutated TβRII is not functional, and they are not responsive to TGF-β treatment (**Supplemental Figure 1C**). Overall, these results confirmed that TGF-β induces EMT in LS411N-TβRII cells and LS513 cells and that DSTYK is upregulated by TGF-β.

### DSTYK Is Required for TGF-β-Induced EMT

**Figure 3:**
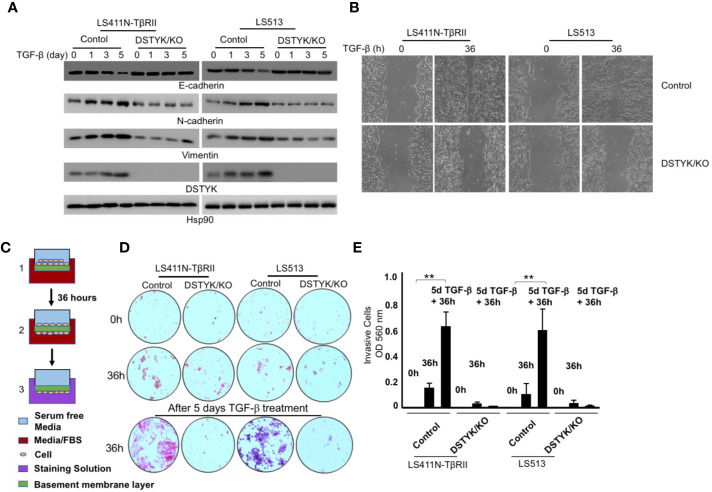
DSTYK is required for TGF-β-induced EMT in CRC cells. **(A)** Immunoblotting analyses were employed to compare the protein levels of DSTYK and the EMT markers E-cadherin, N-cadherin and vimentin between control cells and DSTYK/KO cells during a 5-day TGF-β treatment timecourse. Hsp90 was used as loading control. **(B)** Representative images of a wound healing assay with LS411N-TβRII cells and LS513 cells. **(C)** Schematic of the principle of a cell invasion assay. **(D)** Top: The cells on the basement membrane before invasion at 0h. Middle: The cells migrating to the bottom of membrane after 36h invasion. Bottom: The cells on the bottom of membrane after 36h invasion from 5 days of TGF-β treatment. Representative images of the lower chamber of the trans-well invasion assay in different treatment groups are shown for LS411N-TβRII or LS513 cells. **(E)** The quantification of cell invasion assays in **(D)**. All the experiments were repeated at least three times. **P < 0.01. Data are shown as mean ± SD, n=3 independent experiments.

To test whether DSTYK is indispensable for TGF-β-induced EMT, we employed the Crispr/Cas9 technique to stably knock out DSTYK expression in both LS411N-TβRII and LS513 cells. We treated these two cells with TGF-β for a 5-day time course, and performed an immunoblotting analysis to detect EMT markers (**Figure 3**). We found that there were no significant differences of the protein levels of epithelial marker E-cadherin and mesenchymal markers N-cadherin and vimentin before and after TGF-β treatment in DSTYK/KO cells (**Figure 3A**). Interestingly, DSTYK/KO significantly decreased the TGF-β-mediated migration ability of LS411N-TβRII cells and LS513 cells when compared to control groups, as demonstrated by wound-healing assays (**Figure 3B**). Cell invasion assays were performed using the Matrigel cell invasion assay kit (Cell Biolab, Inc.) to compare the invasive capacity between control and DSTYK/KO cells before and after a 5-day TGF-β treatment. Our results demonstrated that the invasive capability of DSTYK/KO cells is significantly lower than that of control cells after TGF-β treatment (**Figures 3C, D, E**). All the above data suggest that DSTYK is essential for TGF-β-induced EMT and subsequent cell invasion in both LS411N-TβRII cells and LS513 cells.

### TGF-β Induces Apoptosis Instead of EMT in the Absence of DSTYK

**Figure 4:**
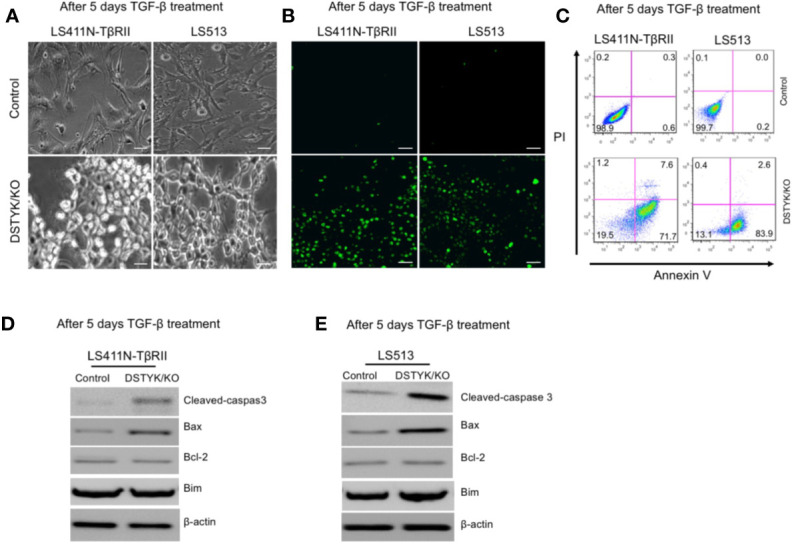
TGF-β induces apoptosis in the absence of DSTYK. **(A)** Morphological differences between control cells and DSTYK/KO cells after 5 days TGF-β treatment. **(B)** TUNEL assays to detect the apoptotic signals in control cells and DSTYK/KO cells after 5 days TGF-β treatment. The green color indicates DNA fragmentation. Scale bar, 100 μm. **(C)** Flow cytometry analysis to compare the levels of cell death by staining with cell surface apoptotic marker annexin V and the necrotic marker PI in control and DSTYK/KO cells after 5 days TGF-β treatment. Western blot analysis of apoptosis-related proteins including cleaved-caspase-3, Bcl-2, Bim, and Bcl2-associated X (Bax) in control and DSTYK/KO LS411N-TβRII cells **(D)** and LS513 cells **(E)** after 5 days TGF-β treatment. β-actin is used as loading control. All experiments were repeated for at least three times, and similar results were observed.

As shown above, in DSTYK/KO cells the protein levels of epithelial markers and mesenchymal markers displayed no significant changes before and after TGF-β treatment. We further characterized the status of these cells after 5 days TGF-β treatment. DSTYK/KO cells not only failed to display a mesenchymal phenotype, but also appeared to enter into a non-proliferative, shrunk state after 5 days TGF-β treatment (**Figure 4A**), suggestive of autophagy, necrosis, or apoptosis. We performed immunoblotting analysis to detect the protein markers for autophagy (LC3B and p62) and for necrosis (cyclophilin A and HMGB1) (15, 16). Immunoblotting results clearly indicated that neither autophagy makers nor necrosis makers demonstrated any significant modulation by TGF-β treatment (data not shown), which excludes the involvement of both autophagy and necrosis. To determine whether apoptosis is induced by TGF-β in DSTYK/KO cells, terminal deoxynucleotidyl transferase dUTP nick end labeling (TUNEL) assays were performed (Jiang et al., 2016). The results demonstrate that strong apoptotic signals were induced in DSTYK/KO cells after 5 days TGF-β treatment (**Figure 4B**). Moreover, flow cytometry analysis was performed to detect the cell surface apoptotic marker annexin V and the loss of plasma membrane integrity (uptake of PI) (Jiang et al., 2016). Our results show that after 5 days TGF-β treatment, there are few annexin V-positive control cells, whereas the majority of DSTYK/KO cells show strong annexin V signals indicative of apoptosis (**Figure 4C**). Interestingly, after 5 days of TGF-β treatment, the level of the anti-apoptotic protein Bcl-2 was not affected, whereas the pro-apoptotic protein Bax was increased in DSTYK/KO cells when compared to control cells. Bim protein level also remained constant (**Figures 4D, E**). Therefore, our results suggest that in the absence of DSTYK, TGF-β may induce apoptosis instead of EMT in CRC cells, and DSTYK may inhibit cell apoptosis through Bax during TGF-β-induced EMT.

### DSTYK Promotes Chemoresistance in CRC Cells

**Figure 5:**
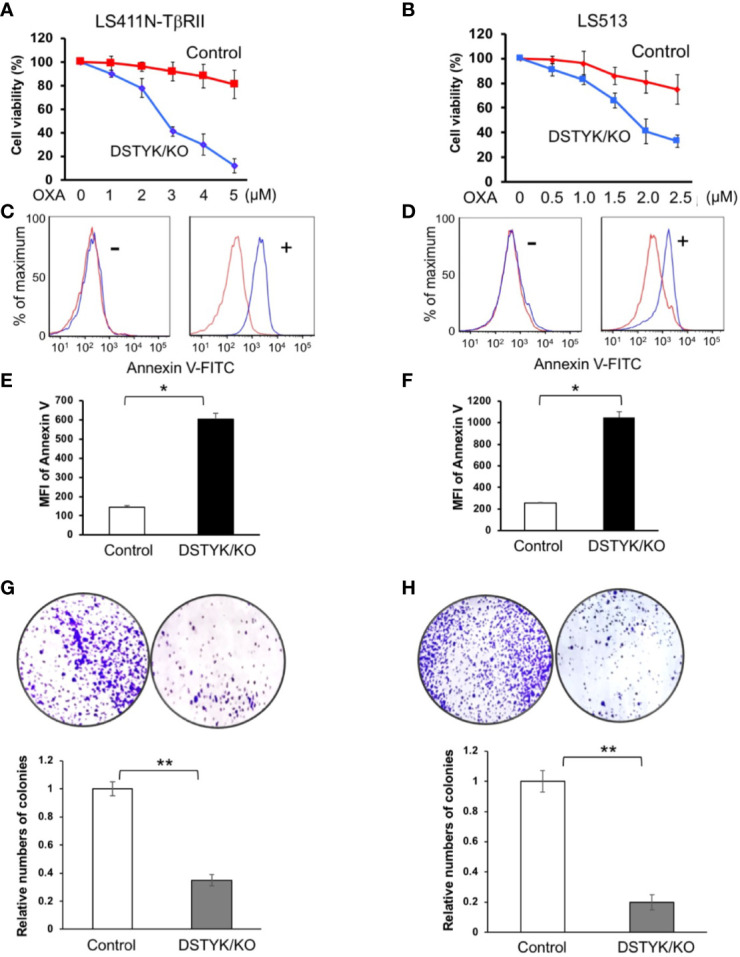
DSTYK inhibits chemotherapeutic agent-induced apoptosis. **(A, B)** MTT assays to compare the chemoresistance between control and DSTYK/KO cells after 3 days OXA treatment. Data are presented as means ± SEM, for n > 3 per dosage point. **(C, D)** Flow cytometry analysis to compare the expression of cell surface apoptotic marker annexin v between control cells and DSTYK/KO cells after a 48 h OXA treatment, 4 µM in **(C)**; 2 µM in **(D)**. Red curves represent control LS411N-TβRII cells or LS513 cells. Blue curves represent DSTYK/KO LS411N-TβRII cells or LS513 cells. ‘-’ means without OXA treatment. ‘+’ means with OXA treatment for 48h. **(E, F)** Quantification of the flow cytometry analyses in **(C, D)**, respectively. **(G, H)** Clonogenic assays and the corresponding quantification results. All experiments were repeated at least three times and similar results were observed. *P < 0.05; **P < 0.01.

Since EMT has been extensively reported to contribute to chemoresistance in chemotherapeutic drug treatment (Fischer et al., 2015; Li et al., 2019), we next performed experiments to assess the role of DSTYK in drug resistance. To test whether DSTYK also plays a role in the apoptosis induced by chemotherapeutic drug treatment in LS411N-TβRII and LS513 cells, we treated both control cells and DSTYK/KO cells with oxaliplatin (OXA) at different concentrations. We noticed that DSTYK/KO cells displayed more chemosensitivity upon chemotherapeutic drug treatment, which is indicated by more apoptosis-mediated cell death in both MTT cell survival assays (**Figures 5A, B**) and flow cytometry analyses with apoptosis marker annexin V (**Figures 5C–F**) and clonogenic assays (**Figures 5G, H**). These data suggest that there is more apoptosis in DSTYK/KO cells treated with OXA than in control cells treated with OXA. In addition, we established the ectopic DSTYK-overexpression (OE) LS411N-TβRII and LS513 cell lines as shown in **Supplemental Figures 2A, B**. We treated both control cells and DSTYK/OE cells with OXA at different concentrations. We found that DSTYK/OE cells display more chemoresistance upon chemotherapeutic drug treatment, which is indicated by MTT cell survival assays (**Supplemental Figures 2C, D**) and flow cytometry analyses with apoptosis marker annexin V (**Supplemental Figures 2E, F**). Therefore, our results further confirm that DSTYK promotes chemoresistance during chemotherapeutic drug treatment in CRC cells.

### Inhibition of DSTYK Expression Enhances Chemotherapy and Attenuates Metastasis in Mouse Model

**Figure 6:**
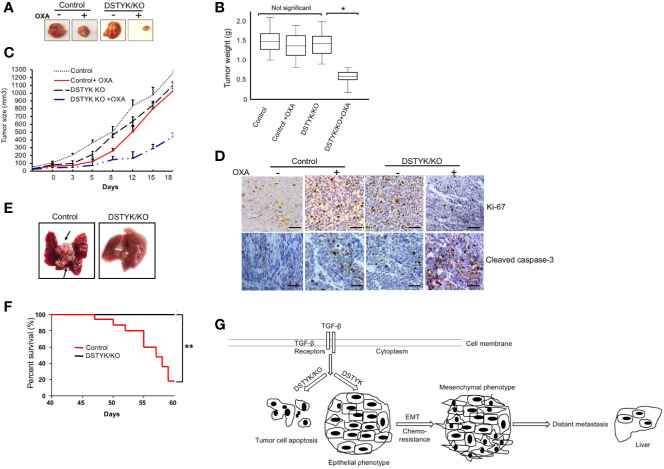
DSTYK knockout facilitates tumor regression after OXA treatment. For each mouse, control LS411N-TβRII cells were implanted into the right flank and DSTYK/KO LS411N-TβRII cells were implanted into the left flank (n=20 per group). After around 3 weeks, mice carrying two tumors of similar size (tumor diameter is around 50 mm as measured with a caliper) started receiving OXA twice weekly. A tumor volume of 1300 mm^3^ was defined as the survival endpoint. Tumors were excised **(A)** and tumors’ weights were evaluated as a box-and-whisker plot **(B)**. Data is presented as mean ± SD. **(C)** The growth curves of control tumors and DSTYK/KO tumors with and without OXA treatment, n=10 per group. **(D)** The levels of Ki-67 and cleaved caspase-3 in tumors tissues from LS411N-TβRII control cells and LS411N-TβRII DSTYK/KO cells detected by immunohistochemical analyses. Scale bar, 50 µm. **(E)** Representative images of liver metastasis (arrows). **(F)** Kaplan-Meier curve assessing the survival of SCID mice injected with LS411N-TβRII control cells or LS411N-TβRII DSTYK/KO cells in cecum wall (n=10/group, 3x). *P < 0.05; **P < 0.01. **(G)** The proposed model for the mechanism underlying the expression and function of DSTYK in TGF-β-induced EMT and chemoresistance.

To provide *in vivo* evidence that DSTYK/KO can attenuate chemoresistance and EMT, we employed both xenograft and orthotopic tumor mouse models. In the xenograft model, 2 × 10^5^ LS411N-TβRII control cells or 2 × 10^5^ DSTYK/KO LS411N-Tβ RII cells were subcutaneously implanted into the flanks of 7- to 8-week-old SCID mice. OXA (10–20 mg/kg) was applied to mice 2 times/week intraperitoneally for 3–4 weeks. After treatments, the size, weight, and growth of tumors from LS411N-TβRII DSTYK/KO cells had regressed significantly more than those tumors from LS411N-TβRII control cells (**Figures 6A–C**), which suggests that DSTYK inhibits chemotherapeutic drug-induced tumor cell death. These results indicate that DSTYK can attenuate the chemosensitivity of tumor cells by inhibiting chemotherapeutic drug-induced apoptosis of tumor cells. Moreover, analyses of IHC revealed that significantly reduced numbers of Ki-67–positive and increased cleaved caspase-3–positive tumor epithelial cells were found in cecal tumors derived from LS411N-TβRII DSTYK/KO cells after OXA treatment when compared to that of control cells (**Figure 6D**). Furthermore, we employed the orthotopic metastatic mouse model in which cells were injected directly into the cecal wall of the SCID mice. DSTYK/KO cells resulted in a significant increase of survival rates and much fewer metastatic lesions in livers and the overall liver tumor burden from LS411N-TβRII DSTYK/KO cells when compared to those of control cells (**Figures 6E, F** and **Table 1**). Similar results were obtained from experiments performed with LS513 control cells and LS513 DSTYK/KO cells as shown in **Supplementary Figure 3** and **Supplemental Table 2**. Collectively, all these results indicate that DSTYK can attenuate the chemosensitivity and promote metastasis of tumor cells by inhibiting chemotherapeutic drug-induced apoptosis and facilitate EMT.

**Table 1:** DSTYK regulates the metastasis of LS411N-TβRII cells implanted in the cecum of SCID mice.

**Treatment group**	**Tumor volume(mm3, mean)**	**Cecum tumor weight (g, mean)**	**Survival**	**Liver metastasis**
**Control**	**1833**	**2.5**	**2/10**	**10/10**
**DSTYK/KO**	**1637**	**2.6**	**10/10**	**0/10**

## Discussion

Accumulated evidence indicates that the EMT is associated with features of advanced cancer progression, including metastasis and chemotherapeutic resistance (Polyak and Weinberg, 2009; Wendt et al., 2009; Mao et al., 2017). However, the mechanism of TGF-β-induced EMT in tumor metastasis and chemotherapeutic resistance remains unclear in CRC. In this study, we describe the functions of DSTYK in human CRC cells and demonstrate that the expression of DSTYK not only is correlated with EMT markers but also related to the survival of CRC patients. Furthermore, we identify DSTYK as a novel effector in TGF-β-induced EMT in CRC cells using both *in vitro* and *in vivo* systems. DSTYK is required for TGF-β-induced EMT and DSTYK knockout *via* Crispr/Cas9 shifts TGF-β-induced EMT to apoptosis. Further, we also characterize the role of DSTYK in chemoresistance during chemotherapeutic drug treatment: DSTYK promotes chemoresistance by inhibiting apoptosis.

We found that DSTYK is upregulated during TGF-β-induced EMT in a gene microarray analysis. Previously, the exact biological role of DSTYK remained unresolved. DSTYK was once identified to be a major determinant of human urinary tract development, downstream of FGF signaling (Sanna-Cherchi et al., 2013). Other studies showed that DSTYK knockdown in zebrafish caused severe developmental defects, suggesting its important role during embryonic development (Sanna-Cherchi et al., 2013). DSTYK homozygous knockout mice exhibited impaired learning and memory capacity compared with that of heterozygous mice (Li et al., 2014). To determine whether DSTYK plays a role in tumorigenesis of colorectal cancer we characterized DSTYK expression in the Gene Expression Omnibus (GEO) and TCGA databases and found that DSTYK is strongly expressed in the cytoplasm of most malignant cells and that the high expression of DSTYK in human colon cancer specimens is associated with tumor progression and/or poor prognosis (Ponten et al., 2011). Although the limited databases concluded that DSTYK is not a prognostic marker in colorectal cancer, the low expression of DSTYK indeed prolongs patients’ life span. Consistent with these findings, we report here that DSTYK protein levels are much higher in human CRC metastatic tumors, including those in lung and liver, than in primary clinical tumor samples of CRC patients. Currently, a lot of factors, inducers and proteins have been identified to be involved in the EMT contributing to metastasis in CRC (Shioiri et al., 2006; Saito et al., 2008; Yan et al., 2015; Weng et al., 2016). In this study we identify a novel element, DSTYK, which plays an important role in TGF-β-induced EMT. Statistically, in our analyses DSTYK is significantly correlated with N-cadherin, vimentin, and TGF-β, but inversely correlated with E-cadherin. We hypothesized that DSTYK, as a novel effector, plays an important role in CRC metastasis and chemoresistance. Therefore, we employed both an *in vitro* cell model and two *in vivo* mouse models to confirm our hypothesis.

Previous reports mostly focused on EMT induced by TGF-β through the canonical Smads signaling pathway in microsatellite stable (MSS) colorectal cancer cell lines, such as HT29 and SW480 (Ahmed et al., 2013; Mouradov et al., 2014). We also observed the upregulation of DSTYK during TGF-β-induced EMT in these cells. However, to generalize our hypothesis, we explored additional cells lines LS411N (microsatellite instable, MSI) and LS513 (MSS). The LS411N cells express a mutated TβRII and are completely unresponsive to TGF-β induction and are impaired in EMT. In contrast, LS513 cells are more sensitive to TGF-β induction and show strong TGF-β-induced EMT. To render LS411N TGF-β competent, we stably overexpressed wild-type TβRII in LS411N cells and established the LS411N-TβRII cell line. As expected, TGF-β not only significantly decreases the epithelial cell marker, E-cadherin, but also increases the mesenchymal markers, N-cadherin and vimentin, in both LS411N-TβRII and LS513 cells. These three EMT makers are commonly used to affirm and characterize the EMT process (Lee et al., 2006). In addition, we find that TGF-β induced the expression of DSTYK transcriptionally. Therefore, our findings provide a novel mechanism for the TGF-β induction of EMT in CRC cells (Pino et al., 2010; Rocha et al., 2018; Yang et al., 2019), and also establish two new CRC cell lines to study TGFβ-induced EMT and metastasis.

Liu et al. reported that TGF-β activated the PI3K/AKT pathway in HCT116 WT-TβRII cells to promote liver metastasis, and a PI3K inhibitor attenuated this effect (Liu et al., 2011). Here we find that after knockout of DSTYK using the Crispr/Cas9 technique, TGF-β treatment failed to induce EMT in CRC cells. Interestingly, we see significant induction of apoptosis instead of EMT in the treated cells when compared to control cells (**Figure 4**). TGF-β–induced EMT is dependent on the expression of DSTYK in colorectal cancer cells. Without DSTYK, TGF-β induces cell apoptosis instead of EMT. Furthermore, chemotherapy is the standard first-line treatment option for CRC patients with metastatic unresectable disease (Van Cutsem et al., 2016; Munker et al., 2018). OXA has been considered as the first‐line chemotherapeutic drug in colorectal cancer treatment (Hsu et al., 2018). Most therapeutic efforts now are focused on validating a novel potential biomarker of tumor progression or response to drug treatment. Our *in vivo* data, from both xenograft and orthotopic mouse models, confirm that DSTYK plays an important role in promoting both tumor metastasis and resistance to OXA therapy. Our findings will aid to the development of therapeutic strategies to overcome CRC metastasis and resistance to chemotherapy. The mechanism we describe here (**Figure 6G**) will enable more efficient therapeutic strategies to improve patient outcomes.

In summary, DSTYK is highly expressed in metastatic CRC; it confers enhanced invasive ability and chemoresistance to CRC cells. DSTYK may be an excellent biomarker for evaluating the significance of CRC metastasis clinically. Targeting DSTYK may provide novel therapeutic approaches for considerably extending the survival rate of CRC patients.

## Conclusion

These studies establish the role of DSTYK in both TGF-β-induced EMT and the chemoresistance in CRC cells. Using engineered cells with modulated DSTYK expression and using an orthotopic mouse model, DSTYK’s mechanism of action and translational significance are elucidated to provide insight into DSTYK’s potential as a therapeutic target for CRC therapy.

## Data Availability Statement

The raw data supporting the conclusions of this article will be made available by the authors, without undue reservation upon reasonable request from qualified researcher.

## Ethics Statement

Written informed consent for participation was not required for this study in accordance with the national legislation and the institutional requirements. The animal study and animal experimental procedures are consistent with our mice protocols that were reviewed and approved by the Institutional Animal Care and Use Committee at ETSU.

## Author Contributions

JZ, ZM, and AT performed most of the experiments and data analyses. JZ drafted the manuscript that was revised by YJ and ZY. ZM, AT, and PM participated in manuscript preparation and discussion. PH and QX gave valuable experimental supports and suggestions. YJ conceived the concept, directed the study, and participated in the manuscript construction.

## Funding

This work was supported by startup funding from East Tennessee State University.

## Conflict of Interest

The authors declare that the research was conducted in the absence of any commercial or financial relationships that could be construed as a potential conflict of interest.
