# Supplementary material for: DSTYK Promotes Metastasis and Chemoresistance via EMT in Colorectal Cancer
Source: Front Pharmacol. 2020 Sep 2;11:1250. doi: 10.3389/fphar.2020.01250 (PMC7493073; doi:10.3389/fphar.2020.01250)
Supplement: Supplementary file 1 [file DataSheet_1.pdf]

Supplemental materials

SUPPLEMENTAL FIGURES AND LEGENDS

SUPPLEMENTAL FIGURE 1.

Effect of TGF-β signaling inhibitor LY2109761 (LY) on the expression of DSTYK. LS411N-TβRII cells (**A**) and LS513 cells (**B**) were treated with TGF-β receptor inhibitor LY (2 μM) followed by treatment with TGF-β (1 ng/ml) for 5 days, before cells were lysed and processed for immunoblotting. (**C**) Immunoblotting to detect the protein levels of DSTYK in LS411N cells containing a mutated, non-functional TβRII during a 5-day TGF-β (1 ng/ml) treatment timecourse. All experiments were repeated at least three times and similar results were observed.

SUPPLEMENTAL FIGURE 2

DSTYK overexpression enhances chemotherapeutic resistance. Western blot analysis of DSTYK protein levels in various clones of DSTYK overexpression (OE) LS411N-TβRII cells (**A**) and LS513 cells (**B**). (**C**) and (**D**) MTT assays to compare the chemoresistance between control and DSTYK/OE cells after 3 days of OXA treatment. Data are presented as mean ± SD, for n= 3 per dosage point. (**E**) and (**F**) Flow cytometry analysis to compare the expression of cell surface apoptotic marker annexin V between control cells and DSTYK/OE cells after a 48 h OXA treatment, 10 μM in (E); 4 μM in (F). Red curves represent control cells and green curves represent DSTYK/OE cells before and after OXA treatment in LS411N-TβRII cells. Red curves represent control cells and dark blue curves represent DSTYK overexpression cells before and after OXA treatment (F) in LS513 cells. '-' means without OXA treatment. '+' means with OXA treatment for 48h. All experiments were repeated at least three times and similar results were observed.

SUPPLEMENTAL FIGURE 3

DSTYK knockout facilitates tumor regression after OXA treatment. For each mouse, control LS513 cells were implanted into the right flank and DSTYK/KO LS513 cells were implanted into the left flank (n=20 per group). After around 3 weeks, mice carrying two tumors of similar size (tumor diameter is around 50 mm as measured with a caliper) were employed. When tumor volume reached around 50 mm^3, OXA was

started twice weekly. Tumor volume of 1300 mm^3 was defined as the survival endpoint. Tumors were excised (**A**), and tumors' weights were evaluated as a box-and-whisker plot (**B**). Data is shown as mean ± SD. (**C**) The growth curves of control tumors and DSTYK/KO tumors with and without OXA treatment (n=10). (**D**) Representative images of liver metastasis (arrows). (**E**) Kaplan-Meier curve assessing survival of SCID mice injected with LS513 control cells or LS513 DSTYK/KO cells in cecum wall (n=10/group, 3x). ** P < 0.01.

SUPPLEMENTAL TABLE 1. The pathologic parameters of patients used for IHC staining of DSTYK in human colorectal primary tumors and secondary tumors.

SUPPLEMENTAL TABLE 2. DSTYK regulates the metastasis of LS513 cells implanted in the cecum of SCID mice.

SUPPLEMENTAL FIGURE 1

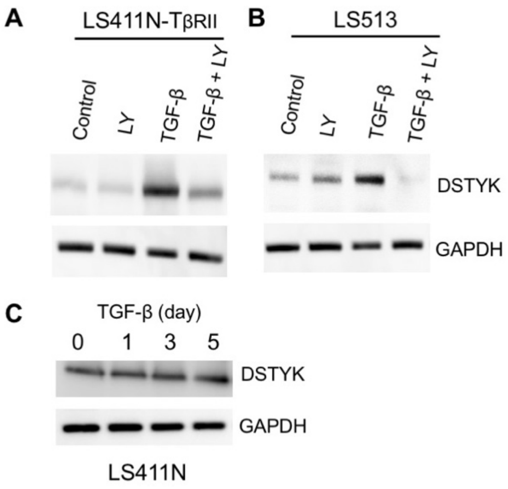

SUPPLEMENTAL FIGURE 2

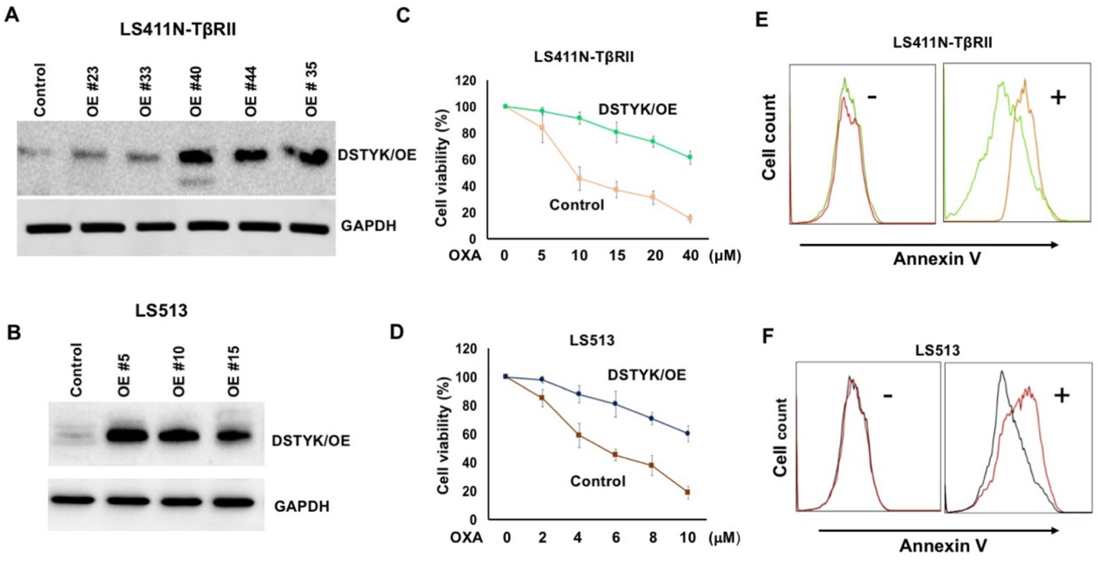

SUPPLEMENTAL FIGURE 3

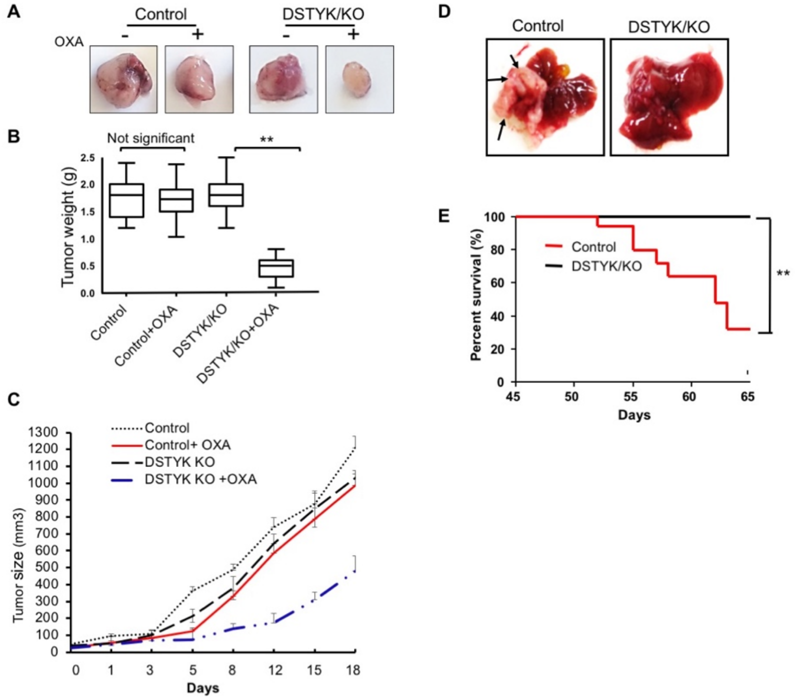

SUPPLEMENTAL TABLE 1

TB#	Race	Sex	Age	Vital Status	Recurrence
**4610**	white	female	50	alive	NO
**4733**	white	male	62	alive	NO
**4818**	white	female	56	alive	NO
**4902**	white	male	89	alive	Since event -no. Colon cancer and surgery before this event
**4915**	white	female	86	alive	NO
**4921**	white	male	63	alive	Patient has never been disease free since diagnosis
**4953**	White	female	59	alive	NO
**5227**	white	male	82	alive	NO
**1776**	white	male	55	alive	NO
**1788**	white	male	40	alive	Unknown
**1858**	white	male	64	dead	Patient has never been disease free since diagnosis
**1872**	black	female	53	alive	NO
**1904**	white	male	57	dead	YES

SUPPLEMENTAL TABLE 2

Treatment group	Tumor volume (mm3 , mean)	Cecum tumor weight (g, mean)	Survival	Liver metastasis
Control	1794.5	2.3	3/10	10/10
DSTYK/KO	1803.7	2.5	10/10	0/10
